# Early Integration of Chinese Medicine Rehabilitation Improves Functional Outcomes in Stroke Patients: A Prospective Pragmatic Study and Nested Case–Control Analysis

**DOI:** 10.1002/cns.71077

**Published:** 2026-08-07

**Authors:** Jialing Zhang, Yan Wa Ng, Keyi Zhou, Hao Ming Fang, Xingyao Wu, Ho Yan Chow, Chin Pong Ho, Zhilin Lin, Chun Hoi Cheung, Linda Ld Zhong, Zhaoxiang Bian

**Affiliations:** ^1^ Vincent V.C Woo Chinese Medicine Clinical Research Institute, School of Chinese Medicine Hong Kong Baptist University Hong Kong China; ^2^ School of Chinese Medicine Hong Kong Baptist University Hong Kong China; ^3^ Biomedical Sciences and Chinese Medicine, School of Biological Sciences Nanyang Technological University Singapore Singapore; ^4^ Chinese EQUATOR Centre Hong Kong China; ^5^ Centre for Chinese Herbal Medicine Drug Development Hong Kong Baptist University Hong Kong China

**Keywords:** acupuncture, Chinese medicine rehabilitation, cognitive function, daily life activity, neurological function, stroke

## Abstract

**Background:**

Chinese medicine (acupuncture and herbal medicine) is widely used in post‐stroke rehabilitation in Southeast Asia. However, evidence on its effectiveness and optimal timing remains inconclusive. This study aimed to determine whether early integration (≤ 6 months post‐stroke) of individualized Chinese medicine rehabilitation (CMR) improves functional outcomes compared with delayed integration (> 6 months) in stroke survivors.

**Methods:**

This prospective pragmatic study enrolled stroke survivors aged 35–90 years from five Hong Kong community centers following hospital discharge. All subjects received a 24‐week individualized CMR scheme comprising acupuncture and Chinese herbal medicine (CHM) granules (administered thrice weekly for 24 weeks, totaling 72 sessions). Outcomes included the changes in activities of daily living (ADL; primary), mini‐mental state examination (MMSE), and traditional Chinese medicine syndrome score scale (TCMSSS). Effectiveness was assessed using linear mixed‐effect models for repeated measures. Multivariate logistic regression identified factors associated with treatment response. In a post hoc nested case–control analysis, early CMR integration (≤ 6 months post‐stroke) was compared with delayed integration (> 6 months) using 1:1 propensity score matching (age, sex, stroke number/type, baseline ADL, hypertension, diabetes, dyslipidaemia, acupuncture sessions, CHM duration).

**Results:**

A total of 398 patients were recruited, of whom 344 (86.6%) completed ≥ 75% of the treatments (≥ 54 sessions). After 24 weeks of treatment, significant improvements were observed in ADL (mean change 5.99, 95% confidence interval [CI] 4.50 to 7.48), MMSE (1.21, 95% CI 0.87 to 1.55), and TCMSSS (−2.89, 95% CI −3.30 to −2.47). Multivariate logistic regression identified baseline ADL dependence (odds ratio [OR] 13.45, 95% CI 7.01 to 25.81), early CMR initiation (OR 2.03, 95% CI 1.23 to 3.35), and lower baseline TCMSSS (OR 0.94, 95% CI 0.91 to 0.98) as independent predictors of response. Kaplan–Meier analysis showed that patients with baseline ADL < 90 had markedly higher responder proportions (46.8% at 60 sessions, 53.6% at 72 sessions) than those with ADL ≥ 90 (9.4% and 12.3%, respectively; log‐rank *P*s < 0.001). Patients who initiated CMR within 6 months had significantly higher responder proportions at 72 sessions (42.9%) compared with those starting later (25.0%; log‐rank *p* = 0.005). Propensity score matching confirmed that early integration yielded superior ADL (mean difference 3.89, 95% CI 1.30 to 6.48; *p* = 0.003) and TCMSSS (−0.86, 95% CI −1.69 to −0.04; *p* = 0.041) outcomes at Week 24. CMR scheme was well‐tolerated, with no treatment‐related adverse events observed.

**Conclusion:**

Initiating CMR within 6 months post‐stroke significantly enhances functional and neurological recovery compared with delayed initiation. These findings support integrating early, individualized CMR into standard stroke rehabilitation protocols.

## Graphical abstract

Initiating Chinese medicine rehabilitation within 6 months post‐stroke improves functional and neurological recovery compared with delayed initiation, increases the probability of meaningful ADL improvement (42.9% vs. 25.0% at 24 weeks), and shows no treatment‐related adverse events.

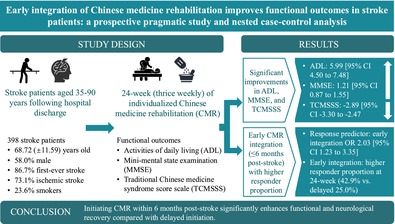

**Abbreviations**
ADL: activities of daily living
CHM: Chinese herbal medicine
CI: confidence interval
CM: Chinese medicine
CMPs: Chinese medicine practitioners
CONSORT: Consolidated Standards of Reporting Trials
MMSE: Mini‐mental state examination
OR: odds ratio
SE: standard error
STRICTA: Standards for Reporting Interventions in Clinical Trials of Acupuncture
TCMSSS: traditional Chinese medicine syndrome score scale

## 1 Introduction

Stroke remains a leading cause of mortality and long‐term disability worldwide, contributing to a substantial global burden of 143 million disability‐adjusted life years (DALYs) annually [1]. With advancements in acute stroke care significantly improving survival rates, stroke has transitioned from a primarily fatal condition to a chronic one characterized by long‐term functional impairments [2, 3]. These impairments, encompassing physical, psychological, and cognitive domains, pose substantial challenges to post‐stroke well‐being and contribute to an increased risk of stroke recurrence [2, 4]. Furthermore, such impairments frequently result in persistent limitations in activities of daily living (ADL) and diminished health‐related quality of life, thereby imposing a significant socioeconomic burden on individuals, families, and healthcare systems [5]. In the Chinese healthcare context, this burden is substantial, with a 2025 retrospective analysis from 22 hospitals in Shenzhen reporting median hospitalization costs of approximately 27,300 CNY (4023 USD) for ischemic stroke and 32,200 CNY (4023 USD) for haemorrhagic stroke [6]. Given the multifaceted impacts of stroke on both individuals and society, expanding rehabilitation management for stroke survivors is essential.

Chinese medicine rehabilitation (CMR), a holistic therapeutic system widely integrated into post‐stroke care across Southeast Asia and Chinese‐speaking regions [7], typically combines acupuncture with syndrome‐differentiated Chinese herbal medicine (CHM) to address the multifaceted sequelae of stroke [8]. Epidemiological estimates indicate that the prevalence of concurrent CMR utilization during post‐stroke rehabilitation ranges from 23% to 66% across different populations and healthcare settings [9, 10]. Acupuncture, a core modality involving the insertion of fine needles at specific acupoints [11], has demonstrated potential in enhancing balance function, reducing spasticity, and improving motor performance, promoting overall well‐being of stroke patients [12, 13], with a favorable safety profile [14]. Concurrently, CHM formulations, characterized by complex compositions and multi‐target mechanisms [15], are posited to exert neuroprotective effects through anti‐inflammatory, antioxidant, and vasodilatory pathways, as well as by enhancing cerebral blood flow, protecting against reperfusion injury, and increasing tissue tolerance to hypoxia [16].

Despite widespread clinical application and biological plausibility of CMR, evidence for its efficacy in stroke rehabilitation remains inconclusive [17]. Systematic reviews have yielded inconsistent findings [18, 19], with most included trials of insufficient quality and sample size to permit definitive conclusions regarding routine clinical application [20]. A methodological limitation across existing studies is substantial variability in CMR initiation timing, which likely represents a significant source of heterogeneity in results. While spontaneous neurological recovery follows a nonlinear trajectory, with most motor gains concentrated within the first 6 months post‐stroke [21], whether this window of heightened neuroplasticity represents the optimal period for initiating adjunctive CMR, and whether early initiation confers incremental benefits beyond spontaneous recovery, remains unverified. Critically, whether CMR initiation timing influences functional outcomes, and whether early integration within this critical window yields superior recovery, has yet to be determined. This prospective pragmatic study with nested case–control analysis evaluated whether early integration (≤ 6 months post‐stroke) of syndrome‐differentiated CMR improves functional outcomes more than delayed integration (> 6 months post‐stroke) in real‐world clinical settings.

## 2 Methods

### 2.1 Design and Setting

We conducted a pragmatic clinical trial (ClinicalTrials.gov Identifier: NCT04971603) in which stroke survivors participated in a 24‐week CMR scheme comprising 72 sessions. The trial was conducted in accordance with the CHM formulas extension for Consolidated Standards of Reporting Trials (CONSORT) [22] and the Standards for Reporting Interventions in Clinical Trials of Acupuncture (STRICTA) [23]. Data were prospectively collected between January 2021 and April 2024. Participants provided voluntary written informed consent after full explanation of the study procedures. Assessments and treatments were conducted at the five neighborhood elderly centres of the Hong Kong Sheng Kung Hui Welfare Council (Data S1) and Chinese medicine clinics of School of Chinese Medicine (SCM), Hong Kong Baptist University (HKBU).

### 2.2 Participants

In the pragmatic clinical trial, patients were eligible if they were: (i) aged 35–90; (ii) diagnosed with cerebral hemorrhage or cerebral infarction through brain computed tomography (CT) or magnetic resonance imaging (MRI); (iii) fulfill the clinical criteria of stroke in ICD‐10 codes of I60, I61, I63, and I64 for subarachnoid hemorrhage, intracerebral hemorrhage, ischaemic stroke, and unspecified stroke [24]; (iv) discharged from hospital; (v) stable vital signs; (vi) and voluntary participation with informed consents signed in this study. Patients were excluded if they had: (i) unconsciousness, aphasia, and cognitive dysfunction; (ii) past history of brain disease (e.g., consciousness disorder due to head trauma, previous brain surgery, spastic disease, or mental illness); (iii) severe heart, liver, or kidney diseases or bleeding disorders; (iv) other serious diseases (e.g., cancer, dementia, Parkinson's disease, and Parkinson's syndrome); (v) participated in other clinical trials within last 3 months; (vi) needle phobia; or (vii) pregnant or lactating female patients. For this study, we included patients received ≥ 2 sessions of CMR.

### 2.3 CMR Scheme

Participants continued to receive their usual treatments independent of CMR scheme and study visits. Participants received syndrome‐differentiated CMR scheme (acupuncture and CHM) thrice weekly for 24 weeks (72 sessions). Registered Chinese medicine practitioners (CMPs) administered the treatments by following the CMR protocol (Data S2). For acupuncture treatments, the 0.25*40 mm needles (Hwato needle, manufactured by Suzhou Hua Tuo Medical Instruments Co. Ltd., China) were used. Principal points included GV20 (Bai Hui), GV26 (Shui Gou), PC6 (Nei Guan), LI11 (Qu Chi), TE5 (Wai Guan), LI4 (He Gu), ST36 (Zu San Li), SP6 (San Yin Jiao), BL40 (Wei Zhong), GB30 (Huan Tiao), GB34 (Yang Ling Quan). For supplementary points, if upper limbs hemiplegia occurred, added TE14 (Jian Liao) and LI10 (Shou San Li); if lower limbs hemiplegia occurred, added GB39 (Xuan Zhong) and LR3 (Tai Chong); if a deviation of the mouth or tongue occurred, added ST4 (Di Cang) and ST6 (Jia Che); additional points were used according to clinical symptoms of participants. For CHM treatments, participants were given Hua Tan Tong Luo granules for pattern of wind and phlegm blocking collaterals; Xing Lou Cheng Qi granules for pattern of phlegm heat; Bu Yang Huan Wu granules for pattern of qi deficiency and blood stasis; and Zhen Gan Xi Feng granules for pattern of stirring wind due to yin deficiency. CHM granules (produced by Purapharm Co. Ltd.) were dispensed weekly by the clinics of SCM, HKBU. The granules were dissolved in 200 mL of hot water and taken twice a day, in the morning and evening after meal.

### 2.4 Measurements

Seven visits were arranged within the 24‐week period (occurring at Week 0, 4, 8, 12, 16, 20 and 24). The primary outcome was the change in activities of daily living (ADL) from baseline to the end of the 24‐week CMR scheme, which was assessed using the Barthel Index and comprises 10 items: feeding, bathing, grooming, dressing, bowels control, bladder control, toilet, transfer, mobility, and stairs [25]. The ADL score ranges from 0 to 100, with a higher score indicating greater ADL independence, while ADL dependency is defined as a score of ≤ 90 [26]. The minimal detectable change in 10 points of ADL was used as a cut‐off score for identifying responders to the CMR scheme [27, 28].

Secondary outcomes included mini‐mental state examination (MMSE) and traditional Chinese medicine syndrome score scale (TCMSSS). MMSE is a widely used cognitive assessment tool and comprises 11 items assessing temporal orientation, spatial orientation, immediate memory, attention/concentration, delayed recall, naming, verbal repetition, reading a sentence, verbal comprehension, writing, and constructional praxis [29]. Its maximum score is 30, with a cut‐off point of 19/20 indicating potential cognitive impairment requiring further evaluation [30]. TCMSSS is developed based on stroke diagnosis and efficacy evaluation criteria [31]. Total scores range from 0 to 52: > 40 indicates very severe neurological impairment, 14–39 indicates moderate to severe impairment, and < 13 indicates mild impairment.

### 2.5 Sample Size Calculation

The sample size was estimated based on anticipated changes in the ADL score. Based on a previous trial on stroke patients receiving acupuncture and CHM, where an ADL score reduction of about 9 was observed [8], we adopted a conservative change of 5 and a standard deviation of 25. With a significance level of 5% and accounting for a 20% drop‐out rate, at least 393 patients were needed to achieve 95% power for detecting differences between baseline and week‐24 ADL scores.

### 2.6 Statistical Analysis

All analyses were performed on the intention‐to‐treat (ITT) population. Descriptive statistics were summarized for baseline variables: continuous data are presented as mean (standard deviations, SD) or median (interquartile ranges) according to distribution, and categorical data as number (percentages). Baseline comparisons were performed using Student's *t*‐test or Mann–Whitney *U* test for continuous variables and Chi‐square or Fisher's exact test for categorical variables.

Linear mixed‐effect models were used to evaluate repeated measurements of ADL, MMSE, and TCMSSS scores over time using the observed data. The models included visits and treatment sessions as fixed effects, with subjects and intercept as random effects; baseline values of the respective outcome were included as covariates. Responders were defined as patients achieving the minimal detectable change in ADL (≥ 10 points) after the 24‐week CMR scheme. Clinical variables were compared between responders and non‐responders, followed by multivariate logistic regression to identify significant predictors of response. Missing data were handled using the last‐observation‐carried‐forward (LOCF) method. Survival analyses were performed to examine the number of treatment sessions required to reach a significant ADL improvement (≥ 10 points). The time‐to‐event variable was defined as the session number at which the improvement was first observed; patients who did not reach the improvement were censored at their last acupuncture session. Kaplan–Meier curves were generated, and a Cox proportional hazards model was built to estimate hazard ratios for predictors of earlier improvement. Subgroup analyses were conducted by baseline ADL status (independent [≥ 90] vs. dependent [< 90]) and by treatment timing (stroke onset ≤ 6 months vs. > 6 months). Survival analyses were performed using R software (“survival” and “survminer” packages).

To compare functional outcomes between early (≤ 6 months post‐stroke) and delayed (> 6 months) integration of CMR, a nested case–control analysis was performed as a post hoc analysis. Patients in the early integration group were matched 1:1 with patients in the delayed integration group using propensity score matching (PSM). Propensity scores were estimated by logistic regression based on baseline covariates: age, gender, number of strokes, type of stroke, ADL baseline score, hypertension, diabetes, dyslipidaemia, completed acupuncture sessions, and received CHM weeks. Nearest‐neighbor matching without replacement was applied with a caliper of 0.2. Balance was assessed by absolute standardized mean difference (SMD), with SMD ≤ 0.2 indicating satisfactory balance. After matching, linear mixed‐effects models were used to compare ADL, MMSE, and TCMSSS scores over time between groups. The models included visits, treatment sessions, group, group‐by‐visit interaction, and baseline values as fixed effects, with subjects and intercepts as random effects, and an unstructured covariance matrix for repeated measurements across visits. Between‐group differences at each visit were estimated from the models. Statistical tests were conducted with SPSS 29.0, R 4.5.2, and Python 3.13.1; a 2‐sided *p‐*value of < 0.05 was considered as statistically significant.

## 3 Results

### 3.1 Clinical Characteristics of Study Subjects

**FIGURE 1:**
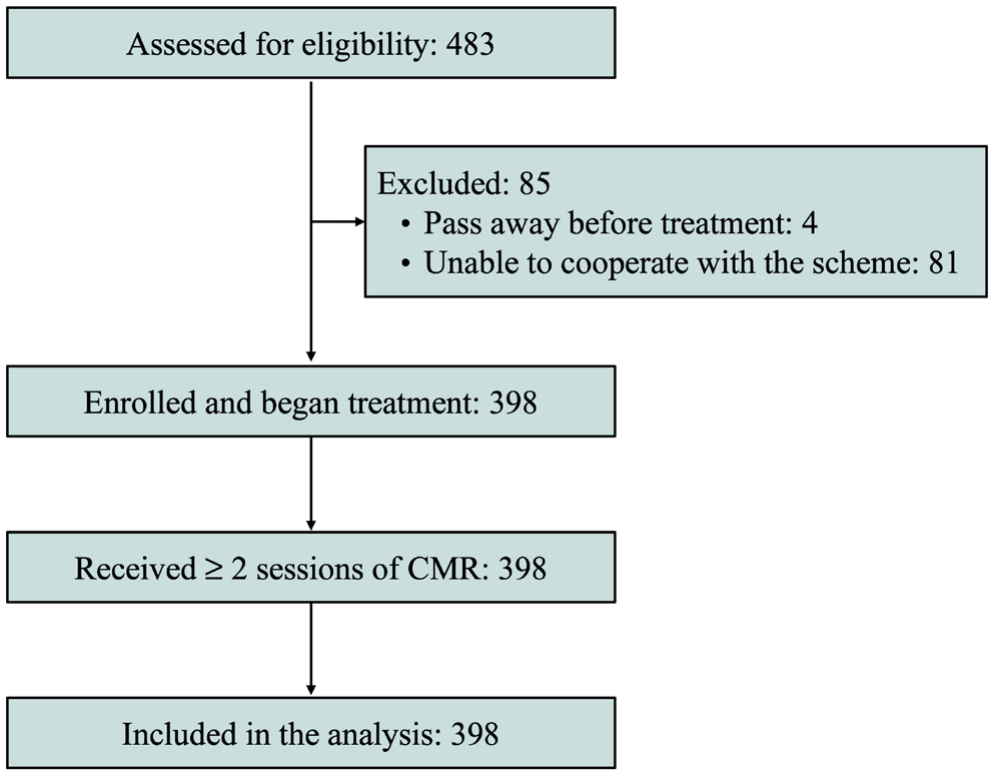
Flow diagram of the study.

During the study period from 2021 to 2024, a total of 398 stroke patients were included (Figure 1). Among them, 344 (86.6%) completed ≥ 75% of the treatments (≥ 54 sessions), and 184 (46.2%) received all 72 sessions. The mean (SD) age was 68.72 (±11.59) years, 58.0% (231/398) were male, 27.9% (111/398) were obese [32], 86.7% (345/398) had first‐ever stroke, 23.6% (94/398) were smokers, 19.1% (76/398) were regular drinker, and the mean interval from stroke onset to CMR integration was 19.07 (±27.30) months (Table 1). Comparisons between responders (112/398; 28.1%) and non‐responders (286/398; 71.9%) showed that responders had lower baseline ADL scores, a smaller proportion of baseline ADL independence, higher baseline TCMSSS scores, a higher prevalence of cardiovascular diseases, and a greater proportion of subjects initiating CMR ≤ 6 months post‐stroke (*P*s < 0.05).

**TABLE 1:** Characteristics of the study subjects.

Characteristics	All (*n* = 398)	Responder (*n* = 112)	Non‐Responder (*n* = 286)	*p* ^a^
Age, year	68.72 ± 11.59	69.02 ± 11.14	68.60 ± 11.78	0.746
Male gender, %	231 (58.0%)	66 (58.9)	165 (57.7)	0.822
Weight, kg	61.62 ± 10.16	61.38 ± 10.11	61.71 ± 10.20	0.773
Height, cm	162.19 ± 8.42	162.04 ± 8.78	162.25 ± 8.29	0.827
BMI, kg/m^2^	23.42 ± 3.32	23.32 ± 3.01	23.46 ± 3.43	0.709
Underweight (< 18.5)	19 (4.8)	7 (6.3)	12 (4.2)	0.344
Normal (18.5–22.9)	184 (46.2)	47 (42.0)	137 (47.9)	
At‐risk of obesity (23.0–24.9)	82 (20.6)	29 (25.9)	53 (18.5)	
Obese (≥ 25.0)	111 (27.9)	29 (25.9)	82 (28.7)	
Refuse to report	2 (0.5)	0 (0.0)	2 (0.7)	
Months after stroke	19.07 ± 27.30	15.18 ± 21.06	20.60 ± 29.28	0.075
≤ 6 months, %^b^	169 (42.6)	59 (52.7)	110 (38.6)	**0.011**
First stroke, %	345 (86.7)	98 (87.5)	247 (86.7)	0.837
Stroke types				0.429
Ischemic stroke, %	291 (73.1)	86 (76.8)	205 (71.7)	
Haemorrhagic Stroke, %	104 (26.1)	26 (23.2)	78 (27.3)	
Refuse to report	3 (0.8)	0 (0.0)	3 (1.0)	
Comorbid diseases				
Hypertension, %	197 (49.5)	60 (53.6)	137 (47.9)	0.309
Hyperlipidaemia, %	88 (22.1)	25 (22.3)	63 (22.0)	0.949
Diabetes, %	66 (16.6)	16 (14.3)	50 (17.5)	0.441
Cardiovascular diseases, %	38 (9.5)	16 (14.3)	22 (7.7)	**0.044**
Liver diseases, %	6 (1.5)	1 (1.7)	5 (0.9)	0.681
Kidney diseases, %	5 (1.3)	1 (0.9)	4 (1.4)	1.000
Smoking habits, %	94 (23.6)	26 (23.2)	68 (23.8)	0.906
Drinking habits, %	76 (19.1)	20 (17.9)	56 (19.6)	0.694
Regular physical exercise (thrice weekly, 30 min/time), %	129 (32.4)	35 (31.3)	94 (32.9)	0.757
ADL baseline score	76.42 ± 27.70	66.16 ± 22.98	80.44 ± 28.38	**< 0.001**
Independent (≥ 90), %	204 (51.3)	21 (18.8)	183 (64.0)	**< 0.001**
MMSE baseline score	19.52 ± 9.15	18.86 ± 9.00	19.78 ± 9.21	0.366
TCMSSS baseline score	9.93 ± 7.00	11.35 ± 6.00	9.37 ± 7.29	**0.011**
Chinese medicine body constitution^c^				
Yang‐deficiency, %	163 (41.0)	45 (40.2)	118 (41.3)	0.844
Yin‐deficiency, %	68 (17.1)	22 (19.6)	46 (16.1)	0.396
Qi‐deficiency, %	143 (35.9)	35 (31.3)	108 (37.8)	0.223
Phlegm‐wetness, %	69 (17.3)	24 (21.4)	45 (15.7)	0.177
Wetness‐heat, %	48 (12.1)	16 (14.3)	32 (11.2)	0.394
Blood‐stasis, %	84 (21.1)	26 (23.2)	58 (20.3)	0.519
Qi‐depression, %	112 (28.1)	34 (30.4)	78 (27.3)	0.538
Special diathesis, %	55 (13.8)	18 (16.1)	37 (12.9)	0.415
Gentleness, %	51 (12.8)	14 (12.5)	37 (12.9)	0.907

*Note:* Data are presented as mean ± SD, or number (%).

Abbreviations: ADL, activities of daily living; MMSE, Mini‐mental state examination; TCMSSS, traditional Chinese medicine syndrome score scale.

^a^ *p* < 0.05, comparisons between responder and non‐responder, calculated with Unpaired *t*‐test, Chi‐square test, or Fisher's exact test.

^b^ One patient with an unclear stroke onset date leaving 397 patients included in this analysis.

^c^ The Chinese medicine body constitution was defined using the Constitution in Chinese Medicine Questionnaire [33].

### 3.2 Improvement in Functional Measurements

As shown in Table 2, there were significant improvements in the ADL, MMSE, and TCMSSS scores from baseline to the end of the 24‐week treatment, with mean changes of 5.99 (95% Confidence Interval [CI] 4.50 to 7.48; Standard Error [SE] = 0.56), 1.21 (95% CI 0.87 to 1.55; SE = 0.13), and −2.89 (95% CI −3.30 to −2.47; SE = 0.16) respectively. Furthermore, the percentage of stroke survivors meeting the criteria for independence (ADL score ≥ 90) exhibited an increase from 51.3% prior to treatment to 63.4% following the 24‐week treatment. Moreover, the items of ADL, MMSE, and TCMSSS exhibited a consistent improvement at each visit (Table S1–S3, Data S3), achieving the highest improvement at week 20 or week 24. Overall missing data number (%) and reasons are in Table S4.

**TABLE 2:** Changes of functional measurements from baseline (*n* = 398).

Timepoint	ADL^a^	MMSE^a^	TCMSSS^b^
Week‐4	0.99 (0.50–1.48)*	0.08 (0.01–0.15)*	−0.23 (−0.41 to −0.05)*
Week‐8	2.02 (1.27–2.77)*	0.41 (0.24–0.59)*	−0.90 (−1.20 to −0.59)*
Week‐12	3.49 (2.38–4.60)*	0.76 (0.49–1.03)*	−1.54 (−1.89 to −1.18)*
Week‐16	4.74 (3.45–6.02)*	0.97 (0.66–1.28)*	−2.04 (−2.41 to −1.67)*
Week‐20	6.08 (4.60–7.56)*	1.21 (0.87–1.55)*	−2.89 (−3.31 to −2.47)*
Week‐24	5.99 (4.50–7.48)*	1.21 (0.87–1.55)*	−2.89 (−3.30 to −2.47)*

*Note:* This table is based on observed data and is not imputed. Data are presented as mean (95% CI).

Abbreviations: ADL, activities of daily living; MMSE, Mini‐mental state examination; TCMSSS, traditional Chinese medicine syndrome score scale.

^a^ A greater positive value represents improvement in symptoms.

^b^ A greater negative value represents improvement in symptoms.

* *p* < 0.001, calculated using a mixed‐effects model with baseline adjustment. Bonferroni correction for multiple comparisons.

### 3.3 Predictors of Treatment Response

Multivariate logistic regression analysis (Table 3) identified 3 independent predictors of treatment response. Participants with baseline ADL < 90 (dependent) were significantly more likely to be responders than those with ADL ≥ 90 (independent) (odds ratio [OR] 13.45, 95% CI 7.01 to 25.81; *p* < 0.001). Early integration of CMR (≤ 6 months post‐stroke) was also associated with higher response likelihood compared with later integration (OR 2.03, 95% CI 1.23 to 3.35; *p* = 0.005). A lower baseline TCMSSS score was associated with increased odds of being a responder (OR 0.94, 95% CI 0.91 to 0.98; *p* = 0.005).

**TABLE 3:** Clinical factors associated with treatment response.

Variables	Unadjusted OR (95% CI)	*p*	Adjusted OR (95% CI)	*p*
ADL < 90 (≥ 90 as reference)	7.70 (4.52–13.11)	< 0.001	13.45 (7.01–25.81)	< 0.001
Stroke onset ≤ 6 months (> 6 months as reference)	1.77 (1.14–2.75)	0.011	2.03 (1.23–3.35)	0.005
TCMSSS baseline score^a^	1.04 (1.01–1.07)	0.012	0.94 (0.91–0.98)	0.005

Abbreviations: ADL, activities of daily living; OR, odds ratio; TCMSSS, traditional Chinese medicine syndrome score scale.

^a^ A greater negative value represents improvement in symptoms.

### 3.4 Number of Treatment Sessions to Reach Significant Improvement

**FIGURE 2:**
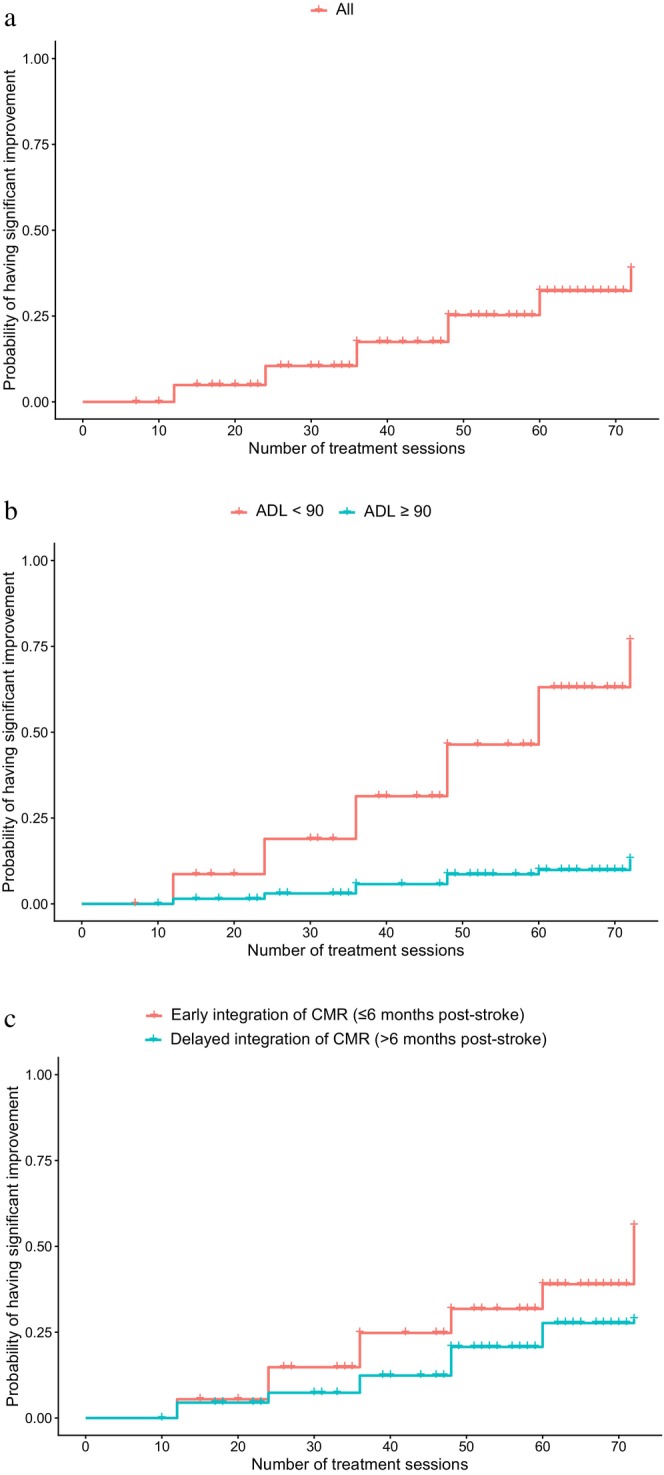
Kaplan–Meier curves for cumulative responder proportion of significant ADL improvement (≥ 10 points) over treatment sessions. (a) The overall cohort; (b) Stratified by baseline ADL status (dependent vs. independent); (c) Stratified by timing of CMR initiation (early vs. delayed).

A total of 28.1% (112/398) of patients achieved a significant improvement in ADL (≥ 10 points; responders). Kaplan–Meier analysis was used to estimate the distribution of the number of treatment sessions until significant ADL improvement. Given that the maximal improvements in ADL, MMSE, and TCMSSS were observed following 60 (week‐20) or 72 sessions (Week‐24), the cumulative responder proportions at these two time points were explored. At 60 treatment sessions, the Kaplan–Meier estimated cumulative responder proportion was 27.6%; at 72 sessions, it increased to 32.2% (Figure 2a). Among patients with baseline ADL < 90 (dependent), the cumulative responder proportion reached 46.8% by 60 sessions and 53.6% by 72 sessions. In contrast, among those with baseline ADL ≥ 90 (independent), the corresponding proportions were 9.4% and 12.3%, respectively (log‐rank *P*s < 0.001; Figure 2b). Stratification by timing of CMR initiation revealed that in the early integration group (≤ 6 months post‐stroke), the cumulative responder proportion was 32.3% at 60 sessions and 42.9% at 72 sessions. In the delayed integration group (> 6 months), the proportions were 24.2% at 60 sessions (log‐rank *p* = 0.051 vs. early) and 25.0% at 72 sessions (log‐rank *p* = 0.005; Figure 2c).

### 3.5 Comparison of Functional Outcomes Between PSM Early and Delayed Integration Groups

**FIGURE 3:**
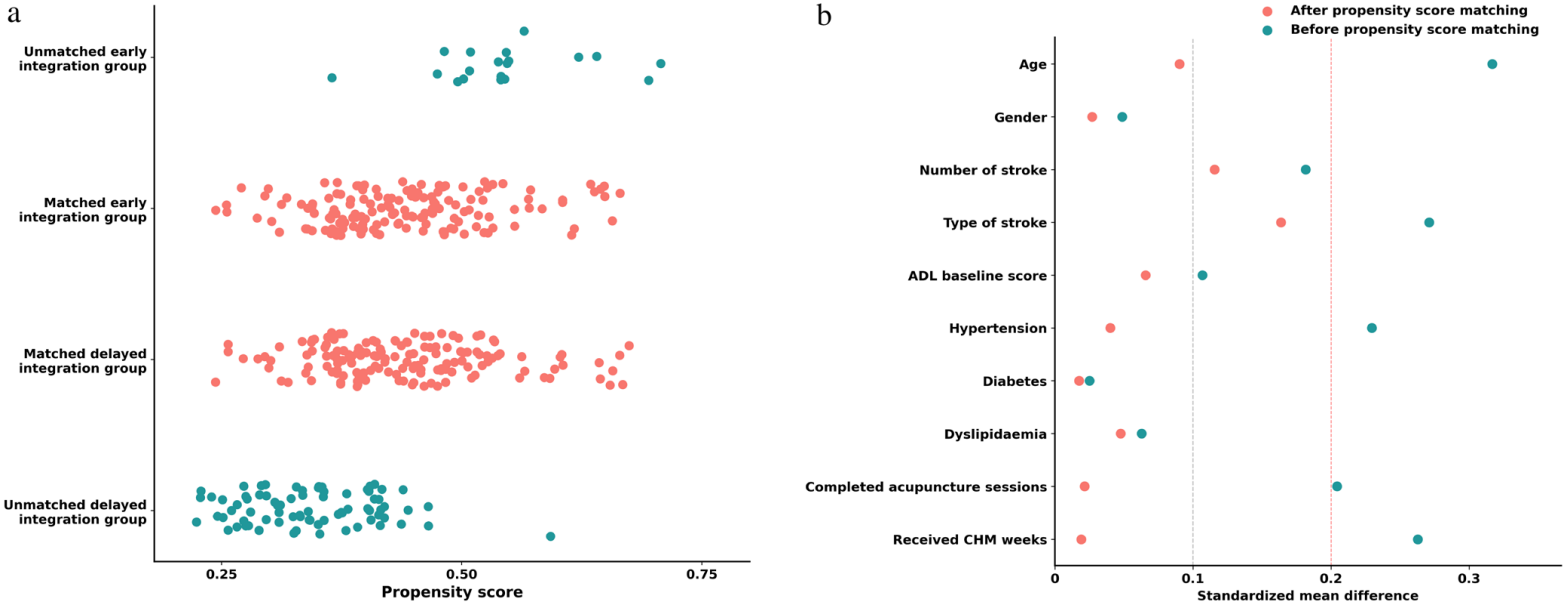
Propensity score distribution and covariate balance after PSM. (a) After 1:1 PSM, both the early and delayed integration groups comprised 150 stroke survivors. (b) SMDs for all covariates fell below the 0.2 threshold.

Using PSM, we successfully matched 150 stroke survivors in the early integration group with 150 survivors in the delayed integration group based on demographic characteristics and completed CMR sessions, forming 150 matched pairs (Figure 3a). The SMDs for all covariates were below 0.2, indicating successful matching (Figure 3b). Post‐matching analysis confirmed balanced baseline characteristics between the matched groups (Table S5). The mean time to CMR initiation post‐stroke was 3.60 (±1.56) months for the early integration group versus 30.55 (±31.44) months for the delayed integration group. Comparison of functional outcomes between the two groups showed significant between‐group differences at week 24 for ADL (3.89, 95% CI 1.30 to 6.48; SE = 1.31; *p* = 0.003) and TCMSSS (−0.86, 95% CI −1.69 to −0.04; SE = 0.42; *p* = 0.041), favoring the early integration group (Table 4). No significant difference was observed for MMSE at week 24 (0.93, 95% CI −0.14 to 2.00; SE = 0.54; *p* = 0.088).

**TABLE 4:** Comparison of functional outcomes between early and delayed CMR integration groups after PSM.

Outcomes	Early integration group (*n* = 150)	Delayed integration group (*n* = 150)	Between‐group difference	*p* ^a^
ADL^b^				
Week‐4	77.92 (76.98–78.86)*	77.39 (76.44–78.34)*	0.53 (−0.36 to 1.42)	0.240
Week‐8	79.60 (78.18–81.01)*	78.32 (76.87–79.77)*	1.28 (−0.06 to 2.62)	0.062
Week‐12	81.57 (79.65–83.49)*	78.68 (76.70–80.67)*	2.89 (1.06 to 4.72)	**0.002**
Week‐16	83.19 (81.06–85.32)*	80.74 (78.52–82.97)*	2.45 (0.40 to 4.51)	**0.020**
Week‐20	84.61 (82.05–87.17)*	81.00 (78.33–83.67)*	3.61 (1.09 to 6.13)	**0.005**
Week‐24	84.93 (82.28–87.58)*	81.04 (78.22–83.86)*	3.89 (1.30 to 6.48)	**0.003**
MMSE^b^				
Week‐4	20.16 (19.80–20.52)	19.99 (19.63–20.36)	0.17 (−0.17 to 0.51)	0.327
Week‐8	21.82 (21.23–22.42)	21.53 (20.91–22.15)*	0.29 (−0.27 to 0.86)	0.307
Week‐12	22.26 (21.40–23.12)*	21.76 (20.87–22.66)*	0.50 (−0.32 to 1.32)	0.231
Week‐16	22.40 (21.47–23.34)*	21.98 (21.01–22.95)*	0.43 (−0.47 to 1.32)	0.351
Week‐20	24.63 (23.58–25.68)*	23.61 (22.52–24.71)*	1.02 (−0.01 to 2.05)	0.053
Week‐24	24.39 (23.29–25.48)*	23.46 (22.29–24.62)*	0.93 (−0.14 to 2.00)	0.088
TCMSSS^c^				
Week‐4	9.34 (9.02–9.65)*	9.52 (9.19–9.85)*	−0.18 (−0.49 to 1.12)	0.233
Week‐8	8.86 (8.35–9.37)*	9.09 (8.57–9.61)*	−0.23 (−0.71 to 2.26)	0.357
Week‐12	8.44 (7.86–9.03)*	8.83 (8.22–9.44)*	−0.39 (−0.95 to 1.72)	0.173
Week‐16	7.99 (7.39–8.58)*	8.26 (7.64–8.89)*	−0.28 (−0.86 to 0.30)	0.344
Week‐20	6.89 (6.21–7.58)*	7.53 (6.82–8.25)*	−0.64 (−1.32 to 0.03)	0.063
Week‐24	6.73 (5.89–7.57)*	7.59 (6.69–8.49)*	−0.86 (−1.69 to −0.04)	**0.041**

*Note:* This table is based on observed data and is not imputed. Data are presented as mean (95% CI).

Abbreviations: ADL, activities of daily living; MMSE, Mini‐mental state examination; TCMSSS, traditional Chinese medicine syndrome score scale.

^a^ *p‐v*alue was calculated using a mixed‐effects model with baseline adjustment to illustrate between‐group differences. Bonferroni correction for multiple comparisons.

^b^ A greater positive value represents improvement in symptoms.

^c^ A greater negative value represents improvement in symptoms.

* *p* < 0.05, calculated using a mixed‐effects model with baseline adjustment to illustrate pre‐ and post‐treatment within‐group differences. Bonferroni correction for multiple comparisons.

### 3.6 Safety

Although three subjects reported COVID‐19 infection, one sustained a rib fracture, and one experienced chest tightness, none of these adverse events (AEs) were considered related to the treatment. No participant discontinued the intervention due to AEs. Acupuncture and CHM were well‐tolerated, and no treatment‐related AEs occurred.

## 4 Discussion

This prospective pragmatic study with a nested case–control analysis investigated whether early integration of CMR within 6 months post‐stroke improves functional outcomes more than delayed initiation in real‐world clinical settings. In the overall cohort, the 24‐week CMR regimen significantly improved ADL, MMSE, and TCMSSS scores over time, reflecting enhancements in daily living ability, cognitive function, and neurological status. Specifically, patients showed significant improvements in overall ADL, as well as in specific areas including feeding, bathing, grooming, dressing, bowel control, toilet use, transfers, mobility, and stairs. The proportion of stroke survivors achieving independence in daily life activities increased from 51.3% to 64.1% after the 24‐week CMR, and 28.1% of patients achieved a clinically meaningful ADL improvement. Moreover, cognitive and neurological functioning, as measured by MMSE and TCMSSS, also showed substantial enhancements. Multivariate logistic regression identified baseline ADL dependence, early CMR initiation, and lower baseline TCMSSS as predictors of response. Kaplan–Meier analysis further demonstrated that patients who initiated CMR within 6 months were more likely to achieve significant ADL improvement. PSM comparison further demonstrated that early integration yielded significantly better ADL and TCMSSS outcomes at week‐24 compared with delayed initiation. No treatment‐related AEs were observed. Collectively, these findings confirm that early CMR within the first 6 months post‐stroke provides incremental functional benefits over delayed CMR initiation, supporting the first 6 months post‐stroke as an optimal period for initiating adjunctive CMR in routine clinical practice.

This trial focused on ADL as the primary outcome, due to the ultimate goal of stroke rehabilitation being to enhance independence in daily activities [34]. In our study, 48.7% of patients were dependent in ADL at baseline, making independence a key rehabilitation objective. The CMR scheme comprised individualized treatments targeting specific impairments: for example, to alleviate dysphagia, needling at GB20 (Feng Chi), GB12 (Wan Gu), and TE17 (Yi Feng) was performed, and prescribing with Jie Yu Dan decoction; to relieve wrist and finger spasticity, needling at LI4 (He Gu) and LI3 (San Jian) was performed; to manage overpronation of the foot, needling at GB40 (Qiu Xu) and KI6 (Zhao Hai) was performed; to improve urinary control, needling at CV3 (Zhong Ji) and CV4 (Guan Yuan) was performed, and prescribing with Tu Si Zi Wan decoction; to enhance spontaneous bowel movements or address fecal incontinence, needling at ST40 (Feng Long) and ST28 (Shui Dao) was performed. These tailored interventions aimed to address specific functional limitations that hinder daily activities. The CMR scheme significantly improved ADL independence with the accumulation of treatment sessions. Among patients with baseline ADL < 90 (dependent), the Kaplan–Meier estimated cumulative responder proportion reached 53.6% at 72 sessions, indicating that over half of initially dependent patients achieved clinically meaningful improvement by the end of 24‐week treatment. Notably, the improvement trend for ADL, MMSE, and TCMSSS appeared to plateau after 20 weeks, as shown by marginal gains between week 20 and 24 in the linear mixed‐model analysis. This plateau may be partly explained by the limited sensitivity of the above measurements across the full spectrum of stroke severity, particularly in mild or severe stroke cases [25, 35], or by a ceiling effect that obscures further functional gains [36]. Whether these indicate that 20 weeks of treatment might be sufficient to meet the rehabilitation needs of stroke patients warrants further investigation in larger studies.

The timing of CMR initiation significantly influenced therapeutic response. Stroke survivors who initiated CMR early (≤ 6 months post‐stroke) were more likely to become responders than those who initiated later, with an adjusted OR of 2.03 (95% CI 1.23 to 3.35). These findings indicate that early integration of CMR confers meaningful advantages over delayed initiation, a conclusion further supported by PSM comparison showing significantly better ADL and TCMSSS outcomes at week‐24 in the early group. Additionally, baseline characteristics were closely associated with the therapeutic response in ADL improvement. Patients with a baseline ADL score < 90 (dependent) were more likely to become responders compared to those with ADL ≥ 90 (OR 13.45, 95% CI 7.01 to 25.81). This finding was not unexpected, as individuals with lower initial ADL scores have greater potential for improvement. Conversely, a higher baseline TCMSSS score (indicating more severe neurological deficits) was associated with a lower likelihood of response (OR 0.94, 95% CI 0.91 to 0.98), consistent with previous research showing that patients with more severe post‐stroke deficits have a poorer prognosis and may benefit less from standard rehabilitation [37, 38]. Such individuals may require a multidisciplinary approach involving multimodal, intensive, and individually tailored interventions [39, 40], and a CMR scheme alone might be insufficient to fully meet their rehabilitation needs. These findings underscore the importance of early intervention, baseline functional status, and neurological deficit severity in optimizing rehabilitation outcomes for stroke patients.

Existing studies on CMR for stroke have several limitations that preclude evidence‐based policy recommendations. First, the optimal timing of CMR initiation remains undetermined [41, 42], leaving clinicians, patients, and carers without guidance on when to start treatment. Second, although individualized Chinese medicine treatments for stroke are increasingly being recognized [43], most trials employ fixed rather than individualized regimens [44], limiting generalizability to real‐world practice. The present study overcomes these limitations by directly comparing early (≤ 6 months) versus delayed (> 6 months) integration using PSM and by adopting a syndrome‐differentiated, individualized CMR protocol. Our finding that early initiation yields superior ADL and TCMSSS outcomes provides evidence to support a clinical guideline recommendation that CMR should be offered within 6 months post‐stroke. Furthermore, the observed plateau after 20 weeks suggests that a 20‐week (60 sessions) course may be sufficient, informing reimbursement policies to avoid unnecessary prolonged treatment. The higher responder probability in the early initiation group (42.9% vs. 25.0% at 72 sessions) highlights the potential for healthcare resource reallocation toward early rehabilitation programs. Collectively, these findings move beyond efficacy evaluation to provide evidence for integrating timely, individualized CMR into standard stroke care pathways.

This study has several strengths. The individualized CMR regimen reflected real‐world clinical practice and enhanced the pragmatic relevance of the findings. Additionally, the prospective pragmatic design with treatment delivered in community elderly centres and Chinese medicine clinics increased the generalisability of the results to routine stroke rehabilitation settings. Furthermore, the use of PSM to create balanced early and delayed integration groups allowed for a rigorous comparison of functional outcomes, thereby reinforcing the credibility of the observed treatment effects. Lastly, the 24‐week treatment period with 72 sessions enabled evaluation of both early and sustained responses, while the inclusion of multiple measurements provided a multidimensional assessment of recovery. Nevertheless, several limitations warrant consideration. Firstly, the study did not include specific assessments of motor function [45]. Given that motor impairment is a core component of post‐stroke disability [46], this omission limits the comprehensiveness of the evaluation. Secondly, the absence of a follow‐up assessment after the 24‐week intervention precludes determination of whether achieved improvements are maintained over time. Thirdly, the nested case–control analysis was performed post hoc; therefore, the findings should be considered exploratory. These limitations constrain the robustness of the conclusions regarding the effectiveness of the CMR scheme. Future research should address these shortcomings to provide a more comprehensive evaluation.

In conclusion, this prospective pragmatic study with a nested case–control analysis demonstrates that early integration of CMR (≤ 6 months post‐stroke) significantly improves ADL and TCMSSS outcomes compared with delayed initiation, with a favorable safety profile. The Kaplan–Meier analysis further showed that patients starting CMR within 6 months had a higher probability of achieving meaningful ADL improvement. These findings support the first 6 months post‐stroke as an optimal window for initiating individualized CMR. However, the absence of specific motor function assessments and post‐treatment follow‐up limits the generalizability of the findings. Future randomized controlled trials with larger sample sizes, long‐term follow‐up, and comprehensive outcome measures are warranted to further validate the effectiveness and safety of early CMR integration in stroke rehabilitation.

## Author Contributions

J.Z. did data analysis and wrote the original draft. Z.B., L.L.Z., and C.H.C. developed and led the study. J.Z., Y.W.N., K.Z., H.M.F., X.W., and H.Y.C. did data curation. Z.L. and C.P.H. did data verification. Y.W.N., K.Z., H.M.F., X.W., and H.Y.C. were responsible for treatments. All authors reviewed and approved the final version of the manuscript.

## Funding

The study was funded by the Bank of China (Hong Kong) (BOCHK), Vincent and Lily Woo Foundation, Andrew Hung Chinese Medicine Clinical Research Fund, and the InnoHK initiative of the Innovation and Technology Commission of the Hong Kong Special Administrative Region Government (ITC RC/IHK/4/7 to Z.X.B). The funding bodies were not involved in study design and conduction, data collection, management, analysis and interpretation.

## Ethics Statement

The study was approved by Research Ethical Committee of the Hong Kong Baptist University (REC/20‐21/0292).

## Conflicts of Interest

The authors declare no conflicts of interest.

## Supporting information


**Data S1:** cns71077‐sup‐0001‐DataS1.docx.

**Data S2:** cns71077‐sup‐0002‐DataS2.docx.

**Data S3:** cns71077‐sup‐0003‐DataS3.docx. **Table S1:** Changes of ADL items from baseline at different timepoints.
**Table S2:** Changes of MMSE items from baseline at different timepoints.
**Table S3:** Changes of TCMSSS items from baseline at different timepoints.
**Table S4:** Overview of the missing data for the primary outcome at follow‐ups.
**Table S5:** Characteristics of study subjects of early (≤ 6 months post‐stroke) and delayed CMR integration (> 6 months post‐stroke) before and after PSM.

## Data Availability

Individual, de‐identified patient data are available at the request of investigators who propose to use the data with a methodologically sound research proposal.
