# Supplementary material for: Early Integration of Chinese Medicine Rehabilitation Improves Functional Outcomes in Stroke Patients: A Prospective Pragmatic Study and Nested Case–Control Analysis
Source: CNS Neurosci Ther. 2026 Aug 7;32(8):e71077. doi: 10.1002/cns.71077 (PMC13450123; doi:10.1002/cns.71077)
Supplement: Supplementary file 1 [file CNS-32-e71077-s001.docx]

**Supplementary Material 1**

**Study Centres**

**Five neighbourhood elderly centres of The Hong Kong Sheng Kung Hui Welfare Council**

(1) Good Shepherd Resource Centre

Address: Shop 93, Block I, Lok Oi Lau, 91 Ko Shan Road, To Kwa Wan, Kowloon

(2) H.K.S.K.H. St. Luke's Settlement Neighbourhood Elderly Centre

Address: Unit B, G/F, Tower 2, Kwun Lung Lau, 20 Lung Wah Street, Kennedy Town, Hong Kong

(3) H.K.S.K.H. Kei Oi Neighbourhood Elderly Centre

Address: Unit A, 2/F, Prince Centre, 70 Tai Po Road, Shamshuipo, Kowloon

(4) H.K.S.K.H. Wong Tai Sin District Elderly Community Centre - Lung Tat Point

Address: Flat 105-108, G/F, Lung Tat House, Lower Wong Tai Sin (I) Estate, Wong Tai Sin, Kowloon

(5) H.K.S.K.H. Tuen Mun District Support Centre - The Gathering

Address: G/F, Leung Yin House, Leung King Estate, Tuen Mun, New Territories

**Three Chinese medicine clinics of School of Chinese Medicine, Hong Kong Baptist University**

(1) Hong Kong Baptist University Mr. & Mrs. Chan Hon Yin Chinese Medicine Specialty Clinic and Good Clinical Practice Centre

Address: 1/F., Jockey Club School of Chinese Medicine Building, 7 Baptist University Road, Kowloon Tong, Kowloon

(2) Hong Kong Baptist University Chinese Medicine Specialty Centre

Address: 3/F., Tsimshatsui Kai Fong Welfare Association Building, 136A Nathan Road, Tsim Sha Tsui, Kowloon

(3) Hong Kong Baptist University Chinese Medicine Specialty Clinic (North Point)

Address: Shop 3-4, Joyous Hub, 3/F The Tanner Hill, 8 Tanner Road, North Point, Hong Kong

**Chinese Medicine Practitioners**

CHAN Ka Ki, CHAN Lok Yi, CHENG Yiu Lung, CHOW Ho Yan, FANG Hao Ming, HU Kai Xi, KWOK Wai Kit, LAM Sze Ki, LAM Tsz Fung, LAU Tsz Yeung, LUI Lok Yin, NG Chiu Fai, NG Lok Yan, NG Yan Wa, TANG Hiu To, WONG Hoi Ki, WONG Lok Tong, WOO Pui San, WU Hsin Ling, WU Xingyao, ZHOU Keyi
