# Supplementary material for: Early Integration of Chinese Medicine Rehabilitation Improves Functional Outcomes in Stroke Patients: A Prospective Pragmatic Study and Nested Case–Control Analysis
Source: CNS Neurosci Ther. 2026 Aug 7;32(8):e71077. doi: 10.1002/cns.71077 (PMC13450123; doi:10.1002/cns.71077)
Supplement: Supplementary file 2 [file CNS-32-e71077-s002.docx]

**Supplementary Material 2**

**CM rehabilitation treatment regimen**

**1. Acupuncture treatment**

| **Acupuncture regimen** | **Detailed procedures** |
| --- | --- |
| **Basic prescription** | |
| **Xing Nao Kai Qiao** | - Acupoints: PC6 (Nei Guan), GV26 (Ren Zhong), SP6 (San Yin Jiao), HT1 (Ji Quan), BL40 (Wei Zhong), LU5 (Chi Ze). - Procedures:  i. PC6 (bilateral): Penetration angle will be perpendicular to the skin surface. Penetration depth will be 1-1.5 Cun. Apply reducing technique by combining needle rotating, lifting and thrusting methods for 1-3 minutes. ii. GV26: Penetration angle will be perpendicular to the skin surface. Apply reducing technique using the heavy sparrow-pecking method until the subject’s ocular region shows signs of lacrimal fluids. iii. SP6 (affected side): Needle at the posterior border of the tibia, with penetration angle of 45 degrees posterior to the skin surface, penetration depth will be 1-1.5 Cun. Apply reinforcing technique by lifting and thrusting methods until the affected lower limb twitches 3 times. iv. HT1 (affected side): Avoiding axillary hair, point of penetration will be 1 Cun inferior of the HT1 acupoint. Penetration angle will be perpendicular to the skin surface. Penetration depth will be 1-1.5 Cun. Apply reducing technique by lifting and thrusting methods until the affected upper limb twitches 3 times. v. LU5 (affected side): Instruct the subject to maintain their humeroulnar joint at 120 degrees flexion, penetration angle will be perpendicular the skin surface. Apply reducing technique by lifting and thrusting methods until the affected forearm or digits twitches 3 times. vi. BL40 (affected side): Have the subject in supine position, the practitioner will fully extend the subject’s knee joint and partially flex the hip joint of the affected side. Penetration depth will be 1-1.5 Cun. Apply reducing technique by lifting and thrusting methods until the affected lower limb twitches 3 times.  - Duration: Retain needles for 30 minutes. |
| **Individualized prescription** | |
| **Wrist and finger spasticity** | - Points: LI4 (He Gu), LI3 (San Jian), EX-UE9 (Baxie). - Procedures:  i. LI4 (affected side). Penetration angle will be directed towards LI3, which is located at the base of the 2nd MTP (metacarpophalangeal joint). Apply reducing technique by lifting and thrusting methods until the digits of the affected dystonia hand extends or the 2nd digit of the hand twitches 3 times. Insert another needle on LI4 of the affected side with the penetration angle directed towards the base of the 1st MTP joint, penetration depth will be 1-1.5 Cun, apply reducing technique by lifting and thrusting methods until the 1st digit of the hand twitches 3 times. ii. EX-UE9 (affected side): Point of penetration will be 1 Cun proximal from the mid-point of the 2-3, 3-4 and 4-5 MTP joints respectively. Angle of penetration will be directed towards the base of the respective MTP joints. Penetration depth will be 1-1.5 Cun. Apply reducing technique by lifting and thrusting methods until the respective digits twitches 3 times.  - Duration: Retain needles for 30 minutes. |
| **Dysphagia** | - Points: GB20 (Feng Chi), GB12 (Wan Gu), TE17 (Yi Feng), posterior pharyngeal wall. - Procedures:  i. GB20, GB12 and TE17 (bilateral): Angle of penetration will be directed towards the laryngeal prominence. Depth of penetration will be 2-2.5 Cun. Apply reinforcing technique by applying mild but high frequency needle rotation method. The rotation angle will be less than 90 degrees and the frequency of the rotation should be between 120-160 times per minute, perform the technique for 1 minute and retain the needle for 30 minutes. ii. Posterior pharyngeal wall: Administer point pricking method on the posterior pharyngeal wall using a 3 Cun filiform needle or a round-sharp needle for bloodletting. |
| **Dysarthria** | - Points: Ex-HN21 (Shang Lian Quan), EX-HN12 (Jin Jin), EX-HN13 (Yu Ye). - Procedures:  i. Ex-HN21: Point of penetration will be the mid-point between the upper border of the hyoid bone and the lower border of the mandible on the midsagittal plane. Angle of penetration will be directed towards the lingual root. Depth of penetration will be 2 Cun. Apply reducing technique by lifting and thrusting methods until the subject experience numbness on the lingual root. Retain the needle for 30 minutes. ii. EX-HN12 and EX-HN13: Lift the tongue and expose sublingual veins with sterilized tongue forceps, then apply bloodletting technique and withdraw 1-3ml of blood from sublingual veins using a three-edged needle. |
| **Over pronation of the foot** | - Points: GB40 (Qiu Xu), KI6 (Zhao Hai). - Procedures:  i. GB40 (affected side): Angle of penetration will be directed towards KI6 on the same side. Carefully insert the needle between the interosseous space within the talocrural joint. Depth of penetration will be 2-2.5 Cun, causing noticeable movements at the skin surface of KI6. Apply reducing technique by using anticlockwise rotation method for left lower extremity and clockwise rotation method for right extremity respectively for 30 seconds. After the reducing technique, retract the needle for 1-1.5 Cun and retain the needle for 30 minutes.  - Duration: Retain needles for 30 minutes. |
| **Constipation** | - Points: ST40 (Feng Long), ST28 (Shui Dao), ST29 (Gui Lai), Wai Shui Dao (1.5 Cun external from ST28) and Wai Gui Lai (1.5 Cun external from ST29). - Procedures:  i. ST40 (bilateral): Angle of penetration will be perpendicular to the skin. Depth of penetration will be 1-1.5 Cun. Apply reducing technique by needle rotation for 1 minute. ii. ST28 (left), ST29 (left), Wai Shui Dao (left) and Wai Gui Lai (Left): Angle of penetration will be perpendicular to the skin. Depth of penetration will be 1.5-3 Cun. Apply reduction technique by needle rotation for 1 minute.  - Duration: Retain needles for 30 minutes. |
| **Declined respiratory function** | - Points: ST11 (Qi She). - Procedures:  i. ST11(bilateral): Depth of penetration will be 1-1.5 Cun. Apply reinforcing technique by needle rotation until normal spontaneous respiration is observed. Retain the needle and reapply the reinforcing technique every 10 minutes. |
| **Urinal retention** | - Points: CV3 (Zhong Ji), BL54 (Zhi Bian). - Procedures:  i. CV3: Angle of penetration will be perpendicular to the skin surface. Depth of penetration will be 1.5-2 Cun. Apply reducing technique with lifting and thrusting method until the subject experiences a distending sensation radiating to the perineum area. ii. BL54 (bilateral): Angle of penetration will be directed towards ST28 (Shui Dao) of the same side. Apply reducing technique by needle rotation until the subject experience a distending sensation radiating to the anterior perineum area.  - Duration: Retain needles for 30 minutes. |
| **Urinal incontinence** | - Points: CV4 (Guan Yuan), CV3 (Zhong Ji), CV2 (Qu Gu). - Procedures:  i. CV4 and CV3: Angle of penetration will be perpendicular to the skin and the depth of penetration will be 1.5-2 Cun. Apply reinforcing technique with breathing method for 1 minute. After the reinforcing technique, perform needle warming moxibustion by attaching a small section of a moxa stick onto the handle of the inserted needle and subsequently igniting it until complete combustion. ii. CV2: Angle of penetration will be perpendicular to the skin. Depth of penetration will be 1-1.5 Cun. Apply balanced reinforcing and reducing technique with needle rotation for 1 minute.  - Duration: Retain needles for 30 minutes. |
| **Ataxia** | - Points: GV16 (Feng Fu), GV15 (Ya Men) or Cervical Ex-B2 (Hua Tuo spine points). - Procedures:  i. GV16 or GV15 (bilateral): Instruct the subject maintain cervical flexion. Administer acupuncture on either GV16 or GV15, with angle of penetration will be directed towards the laryngeal prominence. Gradually insert the needle with vibrating method, advancing no more than 0.5mm with each movement until the subject exhibits signs of tremor or shaking. Promptly retract the needle and do not rotate the needle throughout the acupuncture. This procedure should be limited to 2 times per week. ii. Cervical Ex-B2 (bilateral): Angle of penetration will be perpendicular to the skin surface. Depth of penetration will be 0.3-0.5 Cun. Apply reinforcing technique with needle rotation method. Retain needles for 30 minutes. |
| **Diplopia** | - Points: BL10 (Tian Zhu), BL1 (Jing Ming), EX-HN7 (Qiu Hou). - Procedures:  i. BL10: Angle of penetration will be perpendicular to the skin. Depth of penetration will be 1-1.5 Cun. Apply reinforcing technique with needle rotation method. ii. BL1 and EX-HN7: Angle of penetration will be perpendicular to the skin and depth of 0.3-0.5 Cun. Do not apply any needle manipulation method.  - Duration: Retain needles for 20-30 minutes. |
| **Symptomatic epilepsy** | - Points: PC7 (Da Ling), CV15 (Jiu Wei). - Procedures:  i. PC7 (bilateral): Angle of penetration will be 75 degrees to the skin. Apply reducing technique by rotation method for 1 minute, this is achieved by performing anticlockwise rotation on the left extremity and clockwise rotation on the right extremity, allow the needle to naturally retract and then retain the needle for 30 minutes. ii. CV15: Instruct the subject to hold their head with both hands, elevate their chest and then insert the needle during inhalation. Angle of penetration will be perpendicular to the skin. Depth of penetration will be 1 Cun. Apply balanced reinforcing and reducing technique with rotation method for 30 seconds, retract the needle promptly after the technique. |
| **Glenohumeral joint pain** | - Points: LI15 (Jian Yu), SI9 (Jian Zhen), SI15 (Jian Zhong Shu), TE14 (Jian Wai Shu), Ashi points. - Procedures:  i. LI15, SI9, SI15 and TE14 (affected side): Angle of penetration will be perpendicular to the skin. Depth of penetration will be 1-1.5 Cun. Apply reducing method with needle rotation method for 1 minute for each acupoint. Retain needles for 20-30 minutes.. ii. Ashi points (affected side): Locate Ashi points around the glenohumeral joint region. Apply point pricking method with a three-edged needle for 3-5 times at each Ashi point, then perform flash cupping to assess the bleeding condition. Retain the cup for approximately 5 minutes and draw approximately 5-10ml of blood. |
| **Vascular Dementia** | - Points: GV20 (Bai Hui), EX-HN1 (Si Shen Cong), ST2 (Si Bai), LR3 (Tai Chong). - Procedures:  i. GV20 and EX-HN1: Angle of penetration will be 45 degrees posterior to skin. Depth of penetration will be 0.3-0.5 Cun. Apply balanced reinforcing and reducing technique with needle rotation method for 1 minute. ii. ST2 (bilateral): Angle of penetration will be perpendicular to the skin. Depth of penetration will be 0.8-1 Cun. Apply reinforcing technique with mild (less than 90 degrees) but high frequency (120-160 times/minute) needle rotation method for 1 minute. iii. LR3 (bilateral): Angle of penetration will be perpendicular to the skin. Depth of penetration will be 0.8-1 Cun. Apply reducing technique with needle rotation method, perform anticlockwise rotation on the left extremity and clockwise rotation on the right extremity. Allow the needle to naturally retract and conduct the reducing technique for 1 minute.  - Duration: Retain needles for 30 minutes. |
| **Sleep wake schedule disorder** | - Points: GV23 (Shang Xing), HT7 (Shen Men). - Procedures:  i. GV23: Apply subcutaneous needle insertion from GV23 to GV20 (Bai Hui). Perform reducing technique with needle rotation method. ii. HT7 (bilateral): Angle of penetration will be perpendicular to the skin. Depth of penetration will be 0.3-0.5 Cun. Apply reducing technique with needle rotation method.  - Duration: Retain needles for 30 minutes. |

**2. Chinese Herbal Medicine (CHM) treatment**

| **CHM regimen** | **Details** |
| --- | --- |
| **Basic regimen** | |
| **Stroke affecting the meridians** | - Basic principles:  i. Circulating blood and transforming stasis: Circulating blood and transforming stasis therapy could be used in the treatment of stroke across various stages. Caution should be exercised when applying blood and transforming stasis therapy in patients with blood disorders, recent history of bleeding, or when active bleeding or trauma (such as fractures) is detected during physical examination. ii. Supplementing Qi and circulating blood: The method of supplementing Qi and circulating blood is considered an effective approach in the treatment of stroke, and the formula Bu Yang Huan Wu Decoction is a representative prescription for this purpose. It has become one of the most commonly used prescriptions for the treatment of ischemic stroke.  - Differentiations:  i. ***Wind and phlegm blocking collaterals pattern:***  - Pathogenesis: Liver wind entangled with phlegm, disturbing the meridians and collaterals, causing obstruction in the cerebral vessels and impaired flow in the meridians. - Principle: Transform phlegm and open collaterals. - Formula: Hua Tan Tong Luo decoction with modifications. - Ingredients: *Pinelliae Rhizoma* (processed; Fa Ban Xia; 3-9 gram), *Atractylodis Macrocephalae Rhizoma* (Bai Zhu; 6-12 gram), *Gastrodiae Rhizoma* (Tian Ma; 3-10 gram), *Salviae Miltiorrhizae Radix et Rhizoma* (Dan Shen; 10-15 gram), *Cyperi Rhizoma* (Xiang Fu; 6-10 gram), *Arisaematis Rhizoma* (Dan Nan Xing; 3-6 gram), *Rhei Radix et Rhizom* (processed with alcohol; Jiu Da Huang; 3-15 gram). - Modifications: For patients with abundant yellow-colored phlegm, add *Trichosanthis Fructus* (Quan Gua Lou; 9-15 gram), *Fritillariae Thunbergii Bulbus* (Bei Mu; 5-10 gram), and *Bambusae Concretio Silicea* (Tian Zhu Huang; 3-9 gram). For patients with a dark purplish tongue or petechiae, add *Persicae Semen* (Tao Ren; 5-10 gram), *Carthami Flos* (Hong Hua; 3-10 gram), *Paeoniae Radix Rubra* (Chi Shao; 6-12 gram). For patients with a greasy yellow tongue coating, add *Scutellariae Radix* (Huang Qin; 3-10 gram) and *Gardeniae Fructus* (Zhi Zi; 6-10 gram). For patients with dizziness and headache, add *Chrysanthemi Flos* (Ju Hua; 5-10 gram) and *Prunellae Spica* (Xia Ku Cao; 9-15 gram). - Administration: Oral.  ii. ***Phlegm heat and Fu organ stagnation pattern:***  - Pathogenesis: Phlegm-heat obstructs the middle jiao, causing abnormal ascending and descending of Qi, leading to obstruction of the Fu organs' Qi circulation. - Principle: Transform phlegm and open the Fu organs. - Formula: Xing Lou Cheng Qi decoction or Da Cheng Qi decoction with modifications. - Ingredients: *Trichosanthis Fructus* (Quan Gua Lou; 9-15 gram), *Arisaematis Rhizoma* (Dan Nan Xing; 3-6 gram), *Rhei Radix et Rhizoma* (decoct later; Sheng Da Huang; 3-15 gram), *Natrii Sulfas* (dissolve in decoction; Mang Xiao; 6-12 gram). - Modifications: The dosage of *Rhei Radix et Rhizoma* (unprocessed; Sheng Da Huang; 3-15 gram) and *Natrii Sulfas* (Mang Xiao; 6-12 gram) should be adjusted according to the patient's constitution, ensuring proper bowel movements without excessive dosage. If Xing Lou Cheng Qi decoction fails to promote Fu organ circulation, it can be replaced with Da Chai Hu decoction, or Xing Lou Cheng Qi decoction can be supplemented with *Magnoliae Officinalis Cortex* (Hou Pu; 3-10 gram) and *Aurantii Fructus Immaturus* (Zhi Shi; 3-10 gram) to regulate Qi circulation. For elderly patients with peeled tongue coating, a red or dark red tongue, add *Rehmanniae Radix* (unprocessed, Sheng Di Huang; 10-15 gram), *Ophiopogonis Radix* (Mai Dong; 6-12 gram), and *Scrophulariae Radix* (X*uan She*n; 9-15 gram). - Administration: Oral.  iii. ***Qi deficiency and blood stasis pattern:***  - Pathogenesis: Insufficient righteous Qi, blood stasis, and impaired circulation in the vessels and collaterals. - Principle: Tonify Qi and activate blood. - Formula: Bu Yang Huan Wu decoction with modifications. - Ingredients: *Astragali Radix* (Huang Qi; 9-30 gram), *Angelicae Sinensis Radix* (Dang Gui; 6-12 gram), *Persicae semen* (Tao Ren; 5-10 gram), *Carthami Flos* (Hong Hua; 3-10 gram)*,* *Paeoniae Radix Rubra* (Chi Shao; 6-12 gram), *Chuanxiong* *Rhizoma* (Chuan Xiong; 3-10 gram), *Pheretima* (Di Long; 5-10 gram)*.* - Administration: Oral.  iv. ***Yin deficiency stirring wind pattern:***  - Pathogenesis: Liver and kidney Yin deficiency, Yin unable to restrain Yang, internal wind stirring, Qi and blood in turmoil, affecting the depleted cerebral vessels. - Principle: Nourish Yin and extinguish wind. - Formula: Zhen Gan Xi Feng or Yu Yin Xi Feng decoction with modifications. - Ingredients: *Achyranthis Bidentatae Radix* (Huai Niu Xi; 5-12 gram), Haematitum (unprocessed, decoct first; Zhe Shi; 9-30 gram), *Fossilia Ossis Mastod* (unprocessed, decoct first; Sheng Long Gu; 15-30 gram), *Ostreae Concha* (unprocessed, decoct first; Sheng Mu Li; 9-30 gram), *Testudinis Carapax et Plastrum* (decoct first; Gui Ban; 9-24 gram), *Paeoniae Radix Alba* (Bai Shao; 6-15 gram)*, Scrophulariae Radix* (Xuan Shen; 9-15 gram)*, Asparagi Radix* (Tian Dong; 6-12 gram)*, Toosendan Fructus* (Chuan Lian Zi; 5-10 gram)*, Hordei Fructus Germinatus* (Mai Ya; 10-15 gram), *Artemisiae Scopariae Herba* (Yin Chen; 6-15 gram)*, Glycyrrhizae Radix et Rhizoma* (Gan Cao; 2-10 gram). Or *Rehmanniae Radix* (Da Huang; 3-15 gram), *Corni Fructus* (Shan Yu Rou; 6-12 gram), *Uncariae Ramulus cum Uncis* (decoct later; Gou Teng; 3-12 gram), *Gastrodiae Rhizoma* (Tian Ma; 3-10 gram)*, Salviae Miltiorrhizae Radix et Rhizoma* (Dan Shen; 10-15 gram), *Paeoniae Radix Alba* (Bai Shao; 6-15 gram)*.* - Administration: Oral. |
| **Individualized prescription** | |
| **Hiccups** | - Principle: Regulating stomach function and reducing reflux. - Formula: Da Cheng Qi or Yi Wei decoction with modifications. - Ingredients: *Rhei Radix et Rhizoma* (decoct later; Da Huang; 3-15 gram), *Magnoliae Officinalis Cortex* (Hou Pu; 3-10 gram)*, Aurantii Fructus Immaturus* (Zhi Shi; 3-10 gram)*, Natrii Sulfas* (dissolve in decoction; Mang Xiao; 6-12 gram). *Or Glehniae Radix* (Sha Shen; 5-12 gram)*, Ophiopogonis Radix* (Mai Dong; 6-12 gram), *Rehmanniae Radix* (Sheng Di Huang; 10-15 gram)*, Polygonati Odorati Rhizoma* (Yu Zhu; 6-12 gram)*, Dendrobii Caulis* (Shi Hu; 6-12 gram )*.* - Administration: Oral. |
| **Dysphagia** | - Formula: - Wind phlegm entering the meridians pattern: symptoms include difficulty swallowing, throat phlegm rales, unsatisfactory expectoration, or accompanied by drooling at the corners of the mouth, a greasy white tongue coating, and a wiry and slippery pulse. Jie Yu Dan decoction with modifications. - Qi deficiency and blood stasis pattern: symptoms include difficulty swallowing, pale and yellow complexion, shortness of breath and fatigue, or accompanied by flaccid and weak limbs, a pale purple tongue with petechiae, a thready and hesitant pulse. Bu Yang Huan Wu or Tong Qiao Huo Xue decoction with modifications. - Ingredients:  i. *Typhonii Rhizoma* (Bai Fu Zi; 3-6 gram)*, Acori Tatarinowii Rhizoma* (Shi Chang Pu; 3-9 gram)*, Polygalae Radix* (Yuan Zhi; 3-10 gram)*, Gastrodiae Rhizoma* (Tian Ma; 3-10 gram)*, Scorpio* (Xie Zi; 3-6 gram)*, Notopterygii Rhizoma et Radix* (Qiang Huo; 3-10 gram)*, Arisaematis Rhizoma* (Nan Xing; 3-6 gram)*, Aucklandiae Radix* (Mu Xiang; 3-6 gram)*, Glycyrrhizae Radix et Rhizoma* (Gan Cao; 2-10 gram)*.* ii. *Astragali Radix* (unprocessed; Sheng Huang Qi; 9-30 gram), *Angelicae Sinensis Radix* (Dang Gui; 6-12 gram)*, Persicae Semen* (Tao Ren; 5-10 gram)*, Carthami Flos* (Hong Hua; 3-10 gram)*, Paeoniae Radix Rubra* (Chi Shao; 6-12 gram)*, Chuanxiong Rhizoma* (Chuan Xiong; 3-10 gram)*, Pheretima* (Di Long; 5-10 gram)*.*  - Administration: Oral. |
| **Shoulder-Hand syndrome** | - Principle: Promote blood circulation and unblock the collaterals. - Ingredients: *Aconiti Radix* (Chuan Wu; 1.5-3 gram)*, Aconiti Kusnezoffii Radix* (Cao Wu; 1.5-3 gram)*, Angelicae Sinensis Radix* (Dang Gui; 6-12 gram)*, Chuanxiong Rhizoma* (Chuan Xiong; 3-10 gram)*, Carthami Flos* (Hong Hua; 3-10 gram)*, Mori Ramulus* (Shang Zhi; 9-15 gram)*, Trachelospermi Caulis et Folium* (Luo Shi Teng; 6-12 gram). - Administration: Take 1000-2000ml of the decoction, steam the affected hand with the hot vapours, then wash and apply the slightly warm decoction to the swollen hand and affected limb, apply twice daily. |
| **Urinal Incontinence** | - Formula:  i. Kidney Qi deficiency pattern: symptoms include urinary incontinence, which may even occur during coughing or laughing, fatigue, a pale tongue with a thin coating, and a weak, deep pulse. Tu Si Zi Wan or Suo Quan Wan with modifications. ii. Damp heat downflow pattern: symptoms include frequent urination, burning sensation during urination, and occasional urinary incontinence, a red tongue with a greasy yellow coating, and a thready, slippery, and rapid pulse. Ba Zhen San with modifications.  - Ingredients:  i. *Cuscutae Semen* (Tu Si Zi; 6-12 gram), *Cistanches Herba* (Rou Cong Rong; 6-10 gram), *Ostreae Concha* (Mu Li; 9-30 gram), *Aconiti Lateralis Radix Praeparata* (processed; Zhi Fu Zi; 3-15 gram), *Schisandrae Chinensis Fructus* (Wu Wei Zi; 2-6 gram), *Galli Gigerii Endothelium Coreneum* (Ji Nei Jin; 3-10 gram), *Mantidis Ootheca* (Sang Piao Xiao; 5-10 gram), *Alpiniae Oxyphyllae Fructus* (Yi Zhi Ren; 3-10 gram), *Linderae Radix* (Wu Yao; 6-10 gram), *Dioscoreae Rhizoma* (Shan Yao; 15-30 gram). ii. Plantaginis Semen (Che Qian Zi; 9-15 gram), Polygoni Avicularis Herba (Bian Xu; 9-15 gram), Dianthi Herba (Qu Mai; 9-15 gram), Talcum (Hua Shi; 10-20 gram), Glycyrrhizae Radix et Rhizoma (Gan Cao; 2-10 gram), Rhei Radix et Rhizoma (Da Huang; 3-15 gram), Gardeniae Fructus (Zhi Zi; 6-10 gram), Junci Medulla (Deng Xin; 1-3 gram).  - Administration: Oral. |
