# Supplementary material for: Early Integration of Chinese Medicine Rehabilitation Improves Functional Outcomes in Stroke Patients: A Prospective Pragmatic Study and Nested Case–Control Analysis
Source: CNS Neurosci Ther. 2026 Aug 7;32(8):e71077. doi: 10.1002/cns.71077 (PMC13450123; doi:10.1002/cns.71077)
Supplement: Supplementary file 3 [file CNS-32-e71077-s003.docx]

**Supplementary Material 3**

**eTable 1.** Changes of ADL items from baseline at different timepoints

| **ADL** | **Week-4** | **Week-8** | **Week-12** | **Week-16** | **Week-20** | **Week-24** |
| --- | --- | --- | --- | --- | --- | --- |
| Feeding/10 | 0.01 (-0.07 to 0.19) | 0.03 (-0.06 to 0.11) | 0.05 (-0.05 to 0.15) | 0.16 (0.03 to 0.28) ^#^ | 0.20 (0.06 to 0.34) ^#^ | 0.20 (0.05 to 0.34) ^#^ |
| Bathing/5 | 0.05 (-0.02 to 0.12) | 0.12 (0.05 to 0.19) ^#^ | 0.27 (0.16 to 0.38) ^#^ | 0.37 (0.23 to 0.51) ^#^ | 0.41 (0.26 to 0.56) ^#^ | 0.41 (0.25 to 0.57) ^#^ |
| Grooming/5 | 0.03 (-0.04 to 0.09) | 0.05 (-0.11 to 0.12) | 0.11 (0.02 to 0.19) ^#^ | 0.20 (0.08 to 0.31) ^#^ | 0.24 (0.12 to 0.36) ^#^ | 0.23 (0.11 to 0.35) ^#^ |
| Dressing/10 | 0.16 (0.06 to 0.25) ^#^ | 0.19 (0.08 to 0.29) ^#^ | 0.38 (0.24 to 0.53) ^#^ | 0.39 (0.24 to 0.55) ^#^ | 0.55 (0.36 to 0.74) ^#^ | 0.55 (0.36 to 0.75) ^#^ |
| Bowels control/10 | 0.07 (-0.01 to 0.14) | 0.14 (0.05 to 0.23) ^#^ | 0.21 (0.11 to 0.31) ^#^ | 0.27 (0.15 to 0.38) ^#^ | 0.35 (0.20 to 0.49) ^#^ | 0.33 (0.18 to 0.48) ^#^ |
| Bladder control/10 | 0.10 (0.18 to 0.19) ^#^ | 0.18 (0.09 to 0.27) ^#^ | 0.20 (0.10 to 0.30) ^#^ | 0.27 (0.16 to 0.38) ^#^ | 0.27 (0.11 to 0.43) ^#^ | 0.19 (0.00 to 0.37) ^#^ |
| Toilet/10 | 0.09 (0.01 to 0.17) ^#^ | 0.20 (0.11 to 0.30) ^#^ | 0.31 (0.16 to 0.45) ^#^ | 0.38 (0.22 to 0.54) ^#^ | 0.46 (0.27 to 0.65) ^#^ | 0.43 (0.24 to 0.62) ^#^ |
| Transfer/15 | 0.17 (0.05 to 0.29) ^#^ | 0.29 (0.16 to 0.43) ^#^ | 0.51 (0.36 to 0.67) ^#^ | 0.71 (0.50 to 0.91) ^#^ | 0.99 (0.75 to 1.24) ^#^ | 1.00 (0.75 to 1.24) ^#^ |
| Mobility/15 | 0.23 (0.09 to 0.37) ^#^ | 0.47 (0.33 to 0.62) ^#^ | 0.76 (0.57 to 0.95) ^#^ | 1.07 (0.83 to 1.31) ^#^ | 1.43 (1.16 to 1.70) ^#^ | 1.41 (1.13 to 1.69) ^#^ |
| Stairs/10 | 0.13 (0.05 to 0.26) ^#^ | 0.45 (0.31 to 0.59) ^#^ | 0.79 (0.60 to 0.97) ^#^ | 1.04 (0.82 to 1.27) ^#^ | 1.38 (1.11 to 1.67) ^#^ | 1.41 (1.13 to 1.70) ^#^ |

Note: This table is based on observed data and is not imputed. Data are presented as mean (95% CI).

Abbreviation: ADL, activities of daily living.

**^#^** *P* < 0.05, calculated using a mixed-effects model (with baseline adjustment) to illustrate pre- and post-treatment differences.

**eTable 2.** Changes of MMSE items from baseline at different timepoints

| **MMSE** | **Week-4** | **Week-8** | **Week-12** | **Week-16** | **Week-20** | **Week-24** |
| --- | --- | --- | --- | --- | --- | --- |
| Temporal orientation/5 | 0.05 (-0.21 to 0.11) | 0.29 (0.20 to 0.37) ^#^ | 0.46 (0.34 to 0.59) ^#^ | 0.55 (0.41 to 0.69) ^#^ | 0.70 (0.54 to 0.86) ^#^ | 0.69 (0.53 to 0.85) ^#^ |
| Spatial orientation/5 | 0.03 (-0.03 to 0.09) | 0.12 (0.05 to 0.19) ^#^ | 0.29 (0.18 to 0.39) ^#^ | 0.37 (0.24 to 0.50) ^#^ | 0.50 (0.35 to 0.65) ^#^ | 0.48 (0.33 to 0.63) ^#^ |
| Immediate memory/3 | 0.04 (-0.03 to 0.08) | 0.15 (0.10 to 0.20) ^#^ | 0.29 (0.22 to 0.35) ^#^ | 0.36 (0.28 to 0.43) ^#^ | 0.51 (0.42 to 0.60) ^#^ | 0.51 (0.42 to 0.61) ^#^ |
| Attention&concentration/5 | 0.05 (-0.04 to 0.13) | 0.37 (0.26 to 0.47) ^#^ | 0.54 (0.42 to 0.66) ^#^ | 0.66 (0.52 to 0.80) ^#^ | 1.10 (0.90 to 1.29) ^#^ | 1.14 (0.94 to 1.34) ^#^ |
| Delayed recall/3 | 0.06 (0.01 to 0.11) ^#^ | 0.23 (0.17 to 0.29) ^#^ | 0.36 (0.29 to 0.43) ^#^ | 0.44 (0.36 to 0.52) ^#^ | 0.59 (0.49 to 0.69) ^#^ | 0.58 (0.48 to 0.68) ^#^ |
| Naming/2 | -0.00 (-0.02 to 0.01) | 0.00 (-0.01 to 0.02) | 0.01 (-0.01 to 0.04) | 0.02 (-0.01 to 0.05) | 0.02 (-0.01 to 0.05) | 0.01 (-0.02 to 0.05) |
| Verbal repetition/1 | 0.01 (-0.00 to 0.01) | 0.01 (-0.00 to 0.02) | 0.01 (-0.00 to 0.03) | 0.01 (-0.00 to 0.03) | 0.01 (-0.00 to 0.03) | 0.02 (0.00 to 0.04) ^#^ |
| Reading a sentence/1 | -0.01 (-0.01 to 0.00) | 0.00 (-0.01 to 0.01) | 0.01 (-0.01 to 0.03) | 0.01 (-0.01 to 0.03) | 0.01 (-0.01 to 0.03) | 0.01 (-0.01 to 0.03) |
| Verbal comprehension/3 | 0.00 (-0.03 to 0.03) | 0.01 (-0.02 to 0.03) | 0.03 (-0.01 to 0.05) | 0.05 (0.01 to 0.09) ^#^ | 0.08 (0.03 to 0.14) ^#^ | 0.08 (0.02 to 0.14) ^#^ |
| Writing/1 | -0.01 (-0.02 to 0.01) | 0.01 (-0.01 to 0.02) | 0.01 (-0.01 to 0.03) | 0.03 (0.01 to 0.04) ^#^ | 0.04 (0.01 to 0.06) ^#^ | 0.05 (0.02 to 0.08) ^#^ |
| Constructional praxis/1 | 0.01 (-0.01 to 0.03) | 0.04 (0.02 to 0.06) ^#^ | 0.06 (0.04 to 0.08) ^#^ | 0.09 (0.06 to 0.12) ^#^ | 0.11 (0.07 to 0.15) ^#^ | 0.11 (0.07 to 0.15) ^#^ |

Note: This table is based on observed data and is not imputed. Data are presented as mean (95% CI).

Abbreviation: MMSE, mini-mental state examination.

**^#^** *P* < 0.05, calculated using a mixed-effects model (with baseline adjustment) to illustrate pre- and post-treatment differences.

**eTable 3.** Changes of TCMSSS items from baseline at different timepoints

| **TCMSSS** | **Week-4** | **Week-8** | **Week-12** | **Week-16** | **Week-20** | **Week-24** |
| --- | --- | --- | --- | --- | --- | --- |
| Conscious /9 | -0.05 (-0.21 to 0.13) | -0.30 (-0.39 to -0.21) ^#^ | -0.50 (-0.62 to -0.38) ^#^ | -0.60 (-0.73 to -0.46) ^#^ | -0.69 (-0.84 to -0.54) ^#^ | -0.67 (-0.82 to -0.52) ^#^ |
| Language/6 | -0.04 (-0.08 to -0.03) ^#^ | -0.11 (-0.15 to -0.07) ^#^ | -0.14 (-0.20 to -0.08) ^#^ | -0.20 (-0.28 to -0.13) ^#^ | -0.23 (-0.31 to -0.16) ^#^ | -0.23 (-0.31 to -0.15) ^#^ |
| Facial paralysis&eye/6 | -0.02 (-0.03 to 0.01) | -0.05 (-0.08 to -0.03) ^#^ | -0.09 (-0.12 to -0.06) ^#^ | -0.11 (-0.14 to -0.07) ^#^ | -0.13 (-0.17 to -0.10) ^#^ | -0.12 (-0.16 to -0.08) ^#^ |
| Upper limb paralysis/6 | -0.06 (-0.11 to -0.01) ^#^ | -0.18 (-0.23 to -0.12) ^#^ | -0.28 (-0.35 to -0.22) ^#^ | -0.37 (-0.45 to -0.30) ^#^ | -0.58 (-0.68 to -0.49) ^#^ | -0.63 (-0.73 to -0.52) ^#^ |
| Finger paralysis/5 | -0.03 (-0.07 to 0.02) | -0.10 (-0.14 to -0.05) ^#^ | -0.23 (-0.27 to -0.18) ^#^ | -0.31 (-0.37 to -0.25) ^#^ | -0.52 (-0.61 to -0.44) ^#^ | -0.55 (-0.63 to -0.46) ^#^ |
| Lower limb paralysis/6 | -0.03 (-0.07 to 0.01) | -0.08 (-0.12 to -0.04) ^#^ | -0.11 (-0.17 to -0.06) ^#^ | -0.19 (-0.26 to -0.12) ^#^ | -0.31 (-0.39 to -0.22) ^#^ | -0.31 (-0.40 to -0.23) ^#^ |
| Toe paralysis&other/14 | -0.01 (-0.06 to 0.05) | -0.10 (-0.16 to -0.04) ^#^ | -0.20 (-0.27 to -0.13) ^#^ | -0.28 (-0.36 to -0.20) ^#^ | -0.47 (-0.56 to -0.37) ^#^ | -0.45 (-0.55 to -0.36) ^#^ |

Note: This table is based on observed data and is not imputed. Data are presented as mean (95% CI).

Abbreviation: TCMSSS, traditional Chinese medicine syndrome score scale.

**^#^** *P* < 0.05, calculated using a mixed-effects model (with baseline adjustment) to illustrate pre- and post-treatment differences.

**eTable 4. Overview of the missing data for the primary outcome at follow-ups**

| **Missing data at follow-ups** | **All (n=398)** |
| --- | --- |
| Week 4 | 8 (2.0%) |
| No longer willing to participate | 5 |
| Lost to follow-up | 3 |
| Week 8 | 9 (2.2%) |
| Return to work | 1 |
| Choose other treatments | 1 |
| Perceived improvement | 1 |
| No longer willing to participate | 1 |
| Lost to follow-up | 5 |
| Week 12 | 10 (2.5%) |
| Pass away | 1 |
| Perceived improvement | 1 |
| No longer willing to participate | 2 |
| Lost to follow-up | 6 |
| Week 16 | 12 (3.0%) |
| Perceived improvement | 1 |
| No longer willing to participate | 1 |
| Lost to follow-up | 10 |
| Week 20 | 55 (13.8%) |
| Pass away | 1 |
| Choose other treatments | 1 |
| Perceived improvement | 34 |
| No longer willing to participate | 1 |
| Lost to follow-up | 18 |

**eTable 5.** Characteristics of study subjects of early (≤6 months post-stroke) and delayed CMR integration (>6 months post-stroke) before and after PSM

| **Characteristics** | **Before PSM ^^^** | |  | **After PSM** | |  |
| --- | --- | --- | --- | --- | --- | --- |
|  | **Early (n=169)** | **Delayed (n=228)** | ***P* value^#^** | **Early**  **(n=150)** | **Delayed (n=150)** | ***P* value^#^** |
| Age, year | 70.75±11.05 | 67.14±11.75 | **0.002** | 69.72±10.78 | 70.67±10.35 | 0.435 |
| Male gender, % | 96 (56.8%) | 135 (59.2) | 0.631 | 84 (56.0) | 86 (57.3) | 0.816 |
| Weight, kg | 60.05±9.81 | 62.78±10.28 | **0.008** | 60.25±9.71 | 62.31±10.62 | 0.080 |
| Height, cm | 161.59±8.75 | 162.64±8.16 | 0.217 | 161.91±8.91 | 161.85±8.49 | 0.952 |
| BMI classification |  |  | 0.351 |  |  | 0.243 |
| Underweight (<18.5) | 11 (6.5) | 8 (3.5) |  | 10 (6.7) | 4 (2.7) |  |
| Normal (18.5-22.9) | 82 (48.5) | 102 (44.7) |  | 72 (48.0) | 72 (48.0) |  |
| At-risk of obesity (23.0-24.9) | 35 (20.7) | 47 (20.6) |  | 33 (22.0) | 28 (18.7) |  |
| Obese (≥25.0) | 41 (24.3) | 70 (30.7) |  | 35 (23.3) | 45 (30.0) |  |
| Refuse to report | 0 (0.0) | 1 (0.4) |  | 0 (0.0) | 1 (0.6) |  |
| Number of stroke |  |  | 0.083 |  |  | 0.873 |
| First stroke | 141 (83.4) | 204 (89.5) |  | 126 (84.0) | 129 (86.0) |  |
| Two strokes | 21 (12.4) | 22 (9.6) |  | 20 (13.3) | 19 (12.7) |  |
| ≥Three strokes | 7 (4.2) | 2 (0.9) |  | 4 (2.7) | 2 (1.3) |  |
| Type of stroke |  |  | **0.010** |  |  | 0.424 |
| Ischemic stroke, % | 135 (79.9) | 155 (68.0) |  | 119 (79.3) | 121 (80.7) |  |
| Haemorrhagic Stroke, % | 34 (20.1) | 70 (30.7) |  | 31 (20.7) | 27 (18.0) |  |
| Refuse to report | 0 (0.1) | 3 (1.3) |  | 0 (0.0) | 2 (1.3) |  |
| Comorbid diseases |  |  |  |  |  |  |
| Hypertension, % | 95 (56.2) | 102 (44.7) | **0.024** | 82 (54.7) | 79 (52.7) | 0.728 |
| Hyperlipidaemia, % | 40 (23.7) | 48 (21.1) | 0.535 | 33 (22.0) | 36 (24.0) | 0.681 |
| Diabetes, % | 29 (17.2) | 37 (16.2) | 0.805 | 26 (17.3) | 27 (18.0) | 0.880 |
| Smoking habits, % | 41 (24.3) | 53 (23.2) | 0.814 | 37 (25.3) | 38 (25.3) | 0.894 |
| Drinking habits, % | 25 (14.8) | 51 (22.4) | 0.058 | 23 (15.3) | 33 (22.0) | 0.138 |
| ADL baseline score | 74.79±30.24 | 77.79±25.60 | 0.287 | 75.60±29.77 | 77.47±26.96 | 0.570 |
| MMSE baseline score | 18.72±9.24 | 20.10±9.08 | 0.139 | 19.51±9.13 | 20.08±9.00 | 0.584 |
| TCMSSS baseline score | 9.67±7.48 | 10.14±6.65 | 0.512 | 9.71±7.44 | 9.42±6.43 | 0.721 |
| CMR |  |  |  |  |  |  |
| Initiated post-stroke, months | 3.54±1.57 | 30.59±31.39 | **<0.001** | 3.60±1.56 | 30.55±31.44 | **<0.001** |
| Completed acupuncture sessions | 70.0 (63.0-72.0) | 72.0 (67.0-72.0) | **0.014** | 71.0 (63.8-72.0) | 71.0 (65.0-72.0) | 0.364 |
| Weeks received CHM ^a^ | 24.0 (20.0-24.0) | 24.0 (23.0-24.0) | **0.012** | 24.0 (22.0-24.0) | 24.0 (22.0-24.0) | 0.920 |

Note: Data are presented as mean ± standard deviation, median (interquartile range), or number (%).

**^#^** *P* < 0.05, comparisons between early and delayed integration of CMR, calculated with Unpaired *t*-test, Chi-square test, or Fisher’s exact test. Items of statistical significance were highlighted in bold.

^^^ One patient with an unclear stroke onset date, leaving 397 patients included in this analysis.

^a^ Weekly distribution, 3-day supply per pack.

Abbreviations: ADL, activities of daily living; BMI, body mass index; CHM, Chinese herbal medicine; CMR, Chinese medicine rehabilitation; MMSE, mini-mental state examination; PSM, propensity score matching; TCMSSS, traditional Chinese medicine syndrome score scale.
